# Intraoperative Techniques for Language Mapping in Brain Surgery: A Comparison Between Direct Electrical Stimulation (DES) and Electrocorticography (ECoG)

**DOI:** 10.1002/brb3.70900

**Published:** 2025-09-29

**Authors:** Patricia Silva de Camargo, Giovanna de Oliveira Santos e Souza, Analía Arévalo, Guilherme Lepski

**Affiliations:** ^1^ Institute of Biosciences University of São Paulo São Paulo Brazil; ^2^ Departments of Neurology and Psychiatry, Medical School University of São Paulo São Paulo Brazil; ^3^ Department of Experimental Surgery, Medical School University of São Paulo São Paulo Brazil; ^4^ Department of Neurosurgery Eberhard Karls University Tübingen Germany

**Keywords:** awake brain surgery, brain mapping, direct electrical stimulation, electrocorticography, language mapping

## Abstract

**Purpose:**

The purpose of this overview is to compare Direct Electrical Stimulation (DES) and Electrocorticography (ECoG) techniques, assessing their respective strengths, limitations, and roles in ensuring successful language mapping during awake brain surgeries.

**Method:**

This overview aims to compare two techniques used in intraoperative language mapping during awake brain surgery: Direct Electrical Stimulation (DES) and Electrocorticography (ECoG). By summarizing recent advances in both methods, we highlight their respective mechanisms, applications, and roles in improving surgical outcomes. DES is widely considered the gold standard for cortical brain mapping and is applicable in both awake and anesthetized surgeries for treating epilepsy and brain tumors. In contrast, ECoG involves monitoring the brain's electrical activity with or without direct stimulation, as it provides valuable insight into high gamma activity (70–150 Hz), which is strongly associated with speech production.

**Finding:**

ECoG offers a high‐resolution approach to language mapping by detecting high‐gamma activity, reducing the risk of intraoperative seizures, and serving as a complementary or alternative tool to DES in specific clinical scenarios. While DES continues to be the most reliable technique for identifying functional brain areas, it does carry a higher risk of inducing seizures. Furthermore, recent advancements in ECoG‐based speech decoding and brain–computer interfaces (BCIs) underscore the growing potential of ECoG in restoring communication in patients with severe language impairments, extending its applications beyond surgical mapping.

**Conclusion:**

In conclusion, while DES remains the gold standard for intraoperative language mapping, ECoG is emerging as a promising complementary or alternative technique in some clinical cases. This overview highlights the evolving role of ECoG, particularly in the context of speech decoding and BCIs, offering new possibilities for improving surgical outcomes and postoperative quality of life in patients.

## Graphical abstract

ECoG enables high‐resolution language mapping through high‐gamma activity detection, offering a safer alternative or complement to DES. Emerging applications in speech decoding and brain–computer interfaces highlight ECoG's potential to restore communication in patients with severe language impairments.

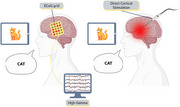

## 1 Introduction

Brain surgery has undergone significant advancements over the past few decades, driven by the development of innovative techniques aimed at improving surgical precision and patient prognosis. Among these approaches, intraoperative stimulation stands out as a crucial tool, with direct electrical stimulation (DES) and electrocorticography (ECoG) providing valuable insights to the neurosurgical team, particularly in the context of language mapping.

DES is currently the gold standard in cortical brain mapping due to its precision and reliability for detecting subcortical eloquent fibers during brain tumor removal (Duffau et al. 2002). This method can be used during awake surgeries for functional mapping, mainly language or perceptual assessment, both in brain tumor resection and epilepsy interventions. In higher‐risk cases, described below, where language is not involved (e.g., motor mapping), it can also be done under anesthesia (Borchers et al. 2011).

An alternative and complementary electrophysiological technique used for language mapping is ECoG, which involves placing a grid of electrodes directly on the cortical surface to monitor electrical activity of the specific brain region without applying direct stimulation (Schalk 2010), but it can also be used for electrical stimulation for identification of epileptogenic areas during surgeries (Chauvel et al. 2019). ECoG is particularly effective for capturing high gamma activity (70–150 Hz), which is strongly associated with speech production, providing both high spatial and temporal resolution (Flinker et al. 2010). The measurement of gamma activity in ECoG makes it an effective tool for identifying critical language areas in the brain (Towle et al. 2008).

In this article, we will explore and compare the two aforementioned techniques, DES and ECoG, analyzing their advantages, limitations, and current impact on the success of language mapping during awake brain surgeries.

## 2 Brain Surgeries and Their Challenges

One of the main challenges in neurosurgery is the preservation of eloquent cortical areas—regions critical for essential functions—while ensuring the success of the intervention and minimizing postoperative impairments. The classic Wada test has long been used to determine the hemispheric lateralization of language and motor functions preoperatively (Baxendale et al. 2008). In addition to this, techniques such as functional magnetic resonance imaging (fMRI) and diffusion tensor tractography (DTI) can provide an overview of regions associated with, but not necessarily responsible for, specific functions, as well as the localization of major tracts (Ferracci and Duffau 2018). However, these imaging methods offer methodological limitations, as they cannot provide direct evidence of the correlation between brain structure and function. They can only indirectly reflect functional organization in the brain—especially with invasion or displacement of axonal tracts by tumors. Currently, the gold standard for the mapping of eloquent areas in neurosurgery is DES of cortical and subcortical regions using electrical currents in order to best account for interindividual differences in brain anatomy (Ferracci and Duffau 2018).

The systematic use of DES can be traced back to the pioneering studies of Penfield and Jasper (1954), “Epilepsy and the Functional Anatomy of the Human Brain,” in which electrical current was used in epileptic patients to localize cognitive functions in the cortex. The Montreal procedure, developed by Penfield, remains the basis for modern awake craniotomy procedures (Rahimpour et al. 2019; Chang et al. 2015). Later, Ojemann sought to expand on these findings by conducting a probabilistic mapping of language functions, such as production and comprehension, using DES (Rahimpour et al. 2019). Since then, researchers have continued to refine the technique not only to identify and preserve eloquent areas during surgery but also to map complex neural networks, such as those involved in language processing (Rahimpour et al. 2019; De Witte et al. 2015; Duffau 2011).

The complexities of brain surgeries, particularly for conditions such as epilepsy and tumors, will be further explored below.

### 2.1 Epilepsy

Epilepsy is a chronic, noncommunicable neurological disease characterized by recurrent seizures affecting approximately 50 million people worldwide (World Health Organization 2024). Seizures are brief episodes of involuntary movements that may affect a specific part of the body or the entire body (Devinsky et al. 2018). In some cases, seizures may also involve a loss of consciousness and a lack of control over bowel or bladder functions (World Health Organization 2024).

Antiseizure drugs (ASDs) are the primary treatment for epilepsy, aiming to stop or reduce them; however, they do not influence the progression of the condition (Devinsky et al. 2018). For individuals with drug‐resistant epilepsy, surgical intervention may be required, where precise resection of specific brain areas is necessary (Thijs et al. 2019). Surgery also provides additional benefits in terms of controlling seizures and enhancing quality of life (QoL), such as lower risk of injury, increased independence, and potentially better life prospects (Picot et al. 2016; Thijs et al. 2019). Before surgery, it is essential to define the epileptogenic zone (the brain area where seizures originate), assess the estimated risks of postsurgical deficits, and predict outcomes (Rosenow 2001). Several neurophysiological tests, including specialized structural magnetic resonance imaging (MRI) and fMRI, tractography, electroencephalography (EEG), magnetoencephalography (MEG), and cortico‐cortical evoked potentials (CCEP), can help identify and localize the epileptogenic zone (Rosenow 2001; Pittau et al. 2012; Yamao et al. 2021). This last technique, CCEP, is used to measure functional connectivity between different brain regions and can be easily performed under general anesthesia (Yamao et al. 2021; Titov et al. 2022).

An additional method for localizing the eloquent brain areas in adults and children is ECoG, which measures cortical high‐gamma modulation (Sinai et al. 2005; Zweiphenning et al. 2022). Pediatric studies using ECoG to guide surgical decisions have demonstrated that it can enable more accurate identification of seizure‐generating regions, allowing for more complete resections and improving the chances of achieving better seizure control after surgery (Greiner et al. 2016; Sun et al. 2020). Moreover, in a pediatric case study, post‐resection ECoG demonstrated the elimination of epileptogenic discharges at both the seizure onset sites and at other areas within the epileptogenic zone (Alotaibi et al. 2022). The child recovered successfully, with no language deficits, and remained seizure‐free (Alotaibi et al. 2022).

When surgery is not feasible or has been ineffective, an alternative treatment option is the implantation of a neurostimulation device, such as an intracranial electrode, which remains implanted for several days to weeks (Lesser et al. 2010). This method involves delivering electrical pulses to peripheral nerves, such as the vagus nerve—the first to be approved for stimulation—or to specific brain areas to help prevent potential seizures. Vagus nerve stimulation, whether through scheduled or open‐loop methods, has been shown to reduce seizure frequency by 50% or more in about one‐third of patients and also improve QoL (Boon et al. 2018; Ryvlin et al. 2014). Intraoperative ECoG‐based language mapping has further contributed to treatment planning by identifying high‐gamma activity associated with language processing prior to resecting the epileptogenic tissue. These studies have detected such activity during tasks including auditory and picture naming (Kambara et al. 2018), verbal fluency (Williams Roberson et al. 2020), and motor control, listening, and visual perception (Gruenwald et al. 2023).

### 2.2 Brain Tumors

Gliomas are among the most common tumors affecting the central nervous system and include several subtypes that generally present a poor prognosis. Surgical intervention is recommended as the primary therapeutic option for these cases (Ferracci and Duffau 2018), as it ensures the best outcome in terms of extent of resection (EOR), overall survival (OS), and QoL. These interventions should ideally be awake craniotomies, with the use of DES for identifying eloquent areas near the tumor (Morshed et al. 2020; Ferracci and Duffau 2018).

DES protocols in tumor cases typically use monopolar or bipolar electrodes, with a pulse frequency of 60 Hz and varying intensity (Szelényi et al. 2010; see Section 4.1 for details). Stimulation is applied to cortical and subcortical regions (nodes or connections) while the patient performs a test aimed to evaluate specific linguistic functions; the eloquence of a region is verified when stimulation temporarily interrupts speech (as verified by altered or incorrect responses). (Morshed et al. 2020). The recommendation for this type of surgery in oncological patients is for individuals with supratentorial tumors, even in the absence of preoperative language deficits. Patients must not exhibit significant aphasia or other major impairments, as this could hinder the proper identification of deficits caused by intraoperative stimulation. Furthermore, it is crucial to establish a relationship of mutual trust between the patient and the medical team, ensuring that the patient is aware of the procedure and remains calm and cooperative after the reversal of anesthesia (Morshed et al. 2020).

In a significant effort to gather the main findings in the field, Ferracci and Duffau (2018) indicate that the use of DES during the resection of both high‐grade and low‐grade gliomas allows for greater EOR, improving prognosis and OS in patients. The authors also postulate that the use of intraoperative electrical stimulation combined with neuropsychological monitoring reduces postoperative morbidity, decreases the risk of permanent neurological damage, and significantly enhances patients' QoL.

Finally, the authors emphasize the importance of considering DES not only in tumors located in the left hemisphere, where the language network is predominantly found in most individuals, but also in tumors in the right hemisphere, as important cognitive functions such as theory of mind, visuospatial cognition, and other language functions, such as nonverbal semantics and prosody, may be impaired during resections in this region (Ferracci and Duffau 2018). Indeed, in a recent study, researchers conducted DES in left language‐dominant patients with tumors in the right hemisphere, finding considerable variability in functional impairments, including speech arrest in 11 out of 15 patients (Prat‐Acín et al. 2021). In light of this, the authors conclude that considering the right hemisphere as a uniform and non‐eloquent structure could neglect its role in important language and cognitive tasks, highlighting the importance of awake craniotomy even in cases where the tumor is not located in the canonical language areas (Prat‐Acín et al. 2021; Ferracci and Duffau 2018).

## 3 Current Intraoperative Language Evaluation Protocols

Current protocols for intraoperative language assessment are typically divided into three stages:

- *Preoperative assessment*: Conducted 24–48 h before surgery, this phase involves a range of tests to evaluate various functions associated with linguistic abilities, such as picture naming, word and pseudoword reading, word and pseudoword repetition, and writing, among others (Morshed et al. 2020). This assessment allows the surgical team to tailor the intraoperative evaluation based on the patient's capabilities by removing tasks where preoperative performance is significantly poor. This stage is essential because imaging techniques, such as fMRI and MEG, are not sensitive enough to determine which brain areas are essential for specific functions versus those that are merely involved (Morshed et al. 2020; Chang et al. 2015).
- *Intraoperative assessment*: Performed under an appropriate anesthetic protocol (see Morshed et al. 2020), this stage involves a battery of tests adapted to the patient's preexisting abilities and tumor location. The choice of tests depends on several factors, including the surgical team's preferences, the availability of standardized tasks, and the tumor's location.
- *Postoperative assessment*: Involves re‐administering the comprehensive preoperative test to monitor for any new deficits and assess potential recovery from previous impairments. If possible considering recovery and hospital/medical plan, postoperative assessment should be conducted shortly after surgery, following medical discharge, and repeated ideally 6 months later for a long‐term follow‐up. Since it is not always possible, the assessment could also be performed several days after discharge.

Regarding intraoperative assessment, there is no single standard protocol in use, as the choice of tests depends on the surgical team, availability of standardized tasks, and tumor location, among others. The most commonly used tasks include naming (Martin‐Monzon et al. 2020; Morshed et al. 2020) and number counting (Bu et al. 2021), followed by tasks such as verb generation and action naming (Martin‐Monzon et al. 2020). Other language functions (reading, repetition, writing, and sentence translation) may also be assessed, with several studies emphasizing the importance of evaluating as many functions as possible during stimulation (Bu et al. 2021; Rofes and Miceli 2014). Furthermore, there has been growing attention to assessing other cognitive functions, such as visual processing, working memory, calculation, and emotional processing, within intraoperative protocols (Bu et al. 2021; Prat‐Acín et al. 2021).

Some studies aim to map the correspondence between cortical and subcortical structures and specific language functions, assisting the surgical team in selecting tests based on the tumor's location. For instance, Middlebrooks et al. (2016) described the most common language impairments observed in areas of the frontal, parietal, and temporal lobes, as well as subcortical regions, categorizing them according to the affected function (semantic, phonological, speech output, and orthographic). However, the authors caution that language difficulties induced by DES can vary significantly across various cortical areas. Chang et al. (2015) provided a current map of language functions as a guide for neurosurgeons, linking several subcortical tracts to specific language errors such as speech arrest, dysarthria, and repetition errors associated with the third superior longitudinal fasciculus (SLF III); syntactic errors related to the arcuate fasciculus (AF), inferior fronto‐occipital fasciculus (IFOF), and uncinate fasciculus (UF); and semantic paraphasias linked to the IFOF (for a more recent, similar approach, see Young et al. 2021).

Despite these advances, a significant challenge in using DES for intraoperative language assessment remains the lack of standardized protocols across different centers. This variability complicates the comparison of results across different groups and makes the selection of appropriate tests somewhat arbitrary. To address this, several initiatives have been undertaken to develop a unified instrument for systematic use in intraoperative settings. One such initiative is the Dutch Linguistic Intraoperative Protocol (DULIP) (De Witte et al. 2015), which proposes a comprehensive language assessment utilizing DES. This includes phonological tasks (e.g., word repetition and phonological odd word out reading), semantic tasks (e.g., semantic odd word out reading, semantic odd word out naming, semantic association, and sentence completion), syntactic tasks (e.g., verb generation and action naming), articulatory tasks (e.g., verbal diadochokinesis), and simple object naming. Additional tasks are also performed outside the stimulation period, such as sentence judgment and fluency tasks, which belong to the preoperative and postoperative assessments. The DULIP has helped map the correspondence between language functions and the cortical and subcortical areas affected by DES, facilitating direct comparisons of results and efficient selection of tests based on tumor location and the patient's difficulties. The protocol has been adapted into other languages, contributing to efforts toward multicenter standardization (for example, see Alves et al. 2021 for the European Portuguese adaptation).

Implanted subdural ECoG grids represent one of the most advanced techniques for decoding human movement, vision, and speech. In patients with epilepsy or severe dysarthria, these implanted electrodes offer a rare opportunity to conduct extended language tests over several days, rather than being limited to intraoperative mapping (Miller et al. 2020; Zheng et al. 2021). For example, language tests applied after electrode implantation, including tasks like auditory repetition, auditory naming, sentence completion, visual reading, and picture naming, have enabled the development of a novel model capable of decoding speech from neural signals and generating synthetic speech (Chen et al. 2024). Additionally, studies on phoneme production aimed to identify and distinguish speech elements have shown that phoneme production involves a sequence of robust and reproducible activity patterns on the cortical surface (Ramsey et al. 2018). These advances are only possible due to the ability to assess language over a prolonged period, beyond the time constraints of intraoperative procedures.

## 4 Intraoperative Stimulation Techniques

Intraoperative techniques for functional brain mapping, such as DES and ECoG, have become essential tools in neurosurgery. While DES involves the stimulation of cortical and subcortical regions, ECoG relies on the recording of cortical electrical activity, and both are valuable tools for brain mapping. ECoG, on the other hand, is a real‐time monitoring technique that records cortical electrical activity directly from the brain, allowing the detection of responses and analysis of potential changes during surgery. Both techniques are complementary, offering an accurate and safe approach to brain mapping, especially language mapping, with crucial applications in the treatment of complex neurological conditions, such as brain tumors and epilepsy. This section will explore the principles, methodologies, and applications of these two techniques, highlighting their importance in improving surgical outcomes and patient safety.

### 4.1 Direct Electrical Stimulation

DES techniques induce alterations in membrane excitability, generating subtle perturbations in cortical or subcortical regions (Mandonnet et al. 2009). Although localized, these perturbations, particularly when applied at the axonal level, propagate through subcircuits, selectively inhibiting components of the network associated with the function under investigation (e.g., phonetics, semantics, or syntax in language assessments), thus serving as an entry point into the broader functional network (Mandonnet et al. 2009). DES provides direct evidence of functional disturbance, in contrast to indirect techniques such as fMRI and MEG (Morshed et al. 2020).

Existing DES protocols can be categorized into biphasic and monophasic stimulation methods. Biphasic stimulation, as described by Ojemann in his seminal work (Ojemann and Whitaker 1978, Morshed et al. 2020), employs bipolar electrodes, with a cathode generating positive phases and an anode generating negative phases. Typically applied at lower frequencies—50 Hz in North America and 60 Hz in Europe—biphasic stimulation involves longer trains of 1–4 s (Roux et al. 2017; Mandonnet et al. 2009; Morshed et al. 2020). The resultant electric field is more localized, offering superior spatial specificity but higher current density (Mandonnet et al. 2009; Ritaccio et al. 2018; Morshed et al. 2020).

Conversely, monophasic stimulation, first described by Taniguchi (Taniguchi et al. 1993; Ritaccio et al. 2018), employs a single‐phase current throughout the pulse duration, with anodal currents applied for cortical mapping and cathodal currents for subcortical mapping. This method uses a monopolar electrode and allows for higher frequencies (200–500 Hz) in shorter train durations, such as the train‐of‐five technique (Taniguchi et al. 1993), which lasts between 10.5 and 18.5 ms (Szelényi et al. 2011; Ritaccio et al. 2018; Riva et al. 2016). The resulting electric field exhibits a homogeneous, radiant distribution, encompassing a broader stimulation area with lower current density (Szelényi et al. 2011; Morshed et al. 2020).

Optimal intensity ranges for DES are still debated, but they typically vary from 2 to 6 mA for cortical mapping (Mandonnet et al. 2009; Morshed et al. 2020) and from 1 to 10 mA for subcortical mapping (Ritaccio et al. 2018; Roux et al. 2017; Szelényi et al. 2011). There is substantial interindividual variability in intensity thresholds (Roux et al. 2017), with certain cortical regions requiring stimulation intensities exceeding 6 mA to elicit positive responses, such as functional language disruption. Higher stimulation intensities are generally associated with more pronounced perturbations, including complete speech arrest (Mandonnet et al. 2009).

White‐matter direct stimulation is performed similarly to cortical DES by adopting either a low frequency (60 Hz) or high frequency (train‐of‐five paradigm) protocol, applied during intraoperative language assessment (Ortiz et al. 2021; Puglisi et al. 2019). White matter stimulation, either bipolar or monopolar, depolarizes the ongoing pathways and thus stimulates or inhibits distant targets, differently from direct cortical stimulation, which has a local inhibitory effect in the area under direct influence of the current dispersion.

Monopolar stimulation has been found to be more effective in eliciting motor‐evoked potentials (MEPs) (Szelényi et al. 2011; Ritaccio et al. 2018). Both biphasic and monophasic techniques have demonstrated efficacy in language mapping (Riva et al. 2016; Ritaccio et al. 2018), although some researchers argue that biphasic stimulation reduces the likelihood of false positives (Mandonnet et al. 2009) and provides greater spatial specificity (Mandonnet et al. 2009; Ritaccio et al. 2018; Morshed et al. 2020), despite carrying a higher risk of ictogenic events (Riva et al. 2016; Morshed et al. 2020). On the other hand, monopolar high‐frequency stimulation has been proposed to generate broader language interference, particularly in semantic and phonological processing (Riva et al. 2016), while posing a lower risk of seizure induction due to its shorter train durations (Riva et al. 2016; Ritaccio et al. 2018). Consequently, high‐frequency monopolar stimulation represents a safer alternative in high‐risk patients, such as those with a history of epileptic activity (Riva et al. 2016). Regardless of the technique employed, continuous monitoring of afterdischarge activity using ECoG remains essential (Mandonnet et al. 2009; Ritaccio et al. 2018).

### 4.2 Electrocorticography

ECoG is a technique that can complement or be a viable alternative for electrical stimulation mapping by localizing eloquent brain areas through measurement of high‐gamma activity. It is an invasive technique that involves the direct application of electrodes to the patient's cerebral cortex to record electrical activity (Schalk 2010). In the landmark study by Crone et al. (1998), ECoG‐based presurgical brain mapping was used to identify the primary motor area, observing an increase in gamma band activity, particularly in the 75–100 Hz range, during movement execution. This innovative approach gained significant attention from researchers in the context of epilepsy surgery due to its advantage of not requiring DES of the brain (Sinai et al. 2005; Kambara et al. 2018). Moreover, sensorimotor areas identified through ECoG demonstrated a high degree of agreement with those identified using DES, confirming the consistency between both methods (Wen et al. 2017). This finding demonstrated the utility of ECoG in preoperative functional cortical mapping and highlighted its ability to map multiple functional networks simultaneously (Wen et al. 2017).

For language mapping, ECoG's direct recording of electrical activity from the cortical surface provides exceptional temporal resolution and spatial specificity, currently the closest window into the brain dynamics underlying speech production (Flinker et al. 2010). In both adult and pediatric epilepsy patients, high‐frequency gamma activity (70–100 Hz) is observed in the frontal, parietal, and temporal perisylvian cortical areas during word listening, recognition, and production, making gamma activity in ECoG useful for identifying eloquent language areas (Towle et al. 2008). One study analyzed ECoG signals produced during word production elicited in response to the presentation of written and spoken words (Flinker et al. 2015). The task elicited high‐frequency power in the ECoG signal (gamma frequencies between 70 and 150 Hz) in the perisylvian language cortex, providing a reliable spectral measure of cortical activation. Areas associated with the auditory cortex (superior temporal gyrus—STG and superior temporal sulcus—STS) Broca's area (pars triangularis and opercularis), as well as the premotor and motor areas, displayed temporal propagation during word articulation. Interestingly, during the initiation of speech, no activation was found in Broca's area. This led the researchers to propose that Broca's area is not the site of articulation itself but a key node responsible for manipulating and routing neural information through large‐scale cortical networks that govern essential components of speech production (Flinker et al. 2015).

## 5 DES Versus ECoG

Like any other method, DES and ECoG each have their advantages and limitations. While electrical stimulation mapping is a powerful tool for localizing brain areas critical for specific behavioral tasks, it carries the risk of stimulation‐induced seizures. In contrast, ECoG offers an alternative method that can provide accurate results with minimal risk of such seizures. Table 1 below outlines the technical and functional aspects of both techniques for language mapping. In it, we included 21 quality studies (e.g., original articles, reviews, commentaries) that implemented and/or described either or both techniques, and this sample comprised studies published between 1978 and 2022.

**TABLE 1:** Descriptive characteristics of DES and ECoG in language mapping.

	**DES** (Sinai et al. 2005; Borchers et al. 2011; Bauer et al. 2013; Arya et al. 2018; Morshed et al. 2018)	**ECoG** (Crone et al. 2006; Formaggio et al. 2013; Flinker et al. 2015; Kambara et al. 2018; Alotaibi et al. 2022)
**Purpose**	Mapping brain functions by directly stimulating specific brain areas (electrodes placed on the cortical surface or deeper structures).	Real‐time monitoring of electrical activity (high‐gamma frequency) via a grid of electrodes placed on the cortical surface (dura mater).
**Application**	Neurosurgery (epilepsy, tumor resection, or brain mapping).	Epilepsy surgery and to monitoring seizure activity and brain function.
**Types of stimulation**	Electrical pulse stimulation (single or repetitive), typically low‐voltage	N/A (primarily used for recording)
**Duration of the stimulation**	Short (milliseconds to seconds) with frequent pauses	Continuous recording (real‐time over long durations)
**Resolution**	High‐resolution localized data in specific brain areas.	High temporal and spatial resolution data for cortical surface activity.
**Patient interaction**	Often awake during stimulation to report sensory and motor changes.	Passive, but could be awake during monitoring.
**Risk of seizures induced by stimulation**	Present	Absent (minimal risk of seizures induced by the brain manipulation or electrical stimulation)
**Analysis time**	Real‐time assessment of function (immediate feedback)	Continuous monitoring (postsurgical analysis of recorded data)
**Cortical sites essentials**	Stimulation targets motor, sensory, and language areas (surface or deep structures)	Grid of electrodes placed on the cortical surface
**Postoperative outcomes**	Real‐time feedback to help avoid functional deficits (e.g., speech issues).	Postoperative analysis helps assess seizure focus and functional brain areas (guide treatment and interventions)

DES is an essential method that uses localized electrical stimulation to identify and preserve vital brain functions during surgery, ensuring that key areas, such as motor, sensory, and language regions, are not damaged. It is also currently the most accurate method for localizing the cortical areas responsible for speech and language comprehension. In contrast, ECoG provides a continuous, real‐time recording of the brain's electrical activity. This method is particularly valuable for long‐term data analysis, such as mapping cortical structures, detecting and localizing seizure foci, and assessing functional brain regions in conditions such as epilepsy.

Although both DES and ECoG are valuable tools for language mapping, they have different goals and capabilities. DES remains the only method that allows causal mapping of both cortical and subcortical language areas. It gives the examiner control over the stimulation process and makes it possible to test a wide range of language functions. The types of errors caused by stimulation also provide specific information about different components of the language system.

On the other hand, ECoG, as a recording‐based method, can show whether an area is active during language processing but does not provide direct evidence of its functional role. It also provides insight into the activation of cortical, but not subcortical, areas. Importantly, ECoG is the only technique that allows for the investigation of the temporal dynamics of language processing—that is, capturing how different components of language unfold over time—which is inaccessible through DES alone. Given these differences, the choice of language tasks should be aligned with the strengths and limitations of each technique. For example, when using ECoG, tasks that emphasize the temporal organization of language processes, such as lexical decision and auditory comprehension, can take advantage of its high temporal resolution. In contrast, DES is better suited for tasks aimed at identifying specific cortical or subcortical regions related to linguistic function, such as object naming or repetition, which allow for a clear disruption when a critical area is stimulated. Choosing tasks that match the sensitivity and specificity of each method capitalizes on their strengths and contributes to a more comprehensive and effective surgical procedure.

### 5.1 Limitations

Although both ECoG and DES are valuable techniques for monitoring and mapping language across different populations, special considerations are required when working with pediatric patients. Due to the lower tolerability of the developing brain, some authors suggest reducing stimulation and current, and adjusting frequency and duration accordingly (Gallantine and Mikati 2009; Kambara et al. 2018; Hyslop and Duchowny 2020). ECoG remains essential for monitoring afterdischarges during stimulation given the heightened susceptibility of this group (Gallantine and Mikati 2009; Hyslop and Duchowny 2020; Reecher et al. 2024). Additionally, the immature cortical structure and greater anatomical variability in this population should be considered when determining stimulation sites (Kambara et al. 2018; Reecher et al. 2024). Finally, appropriate strategies should be implemented to ensure the child can effectively cooperate with the surgical team in what is often a highly stressful setting (Kambara et al. 2018; Hyslop and Duchowny 2020).

In adult populations, some challenges should also be acknowledged. The inherently stressful nature of awake surgeries may lead patients to struggle with following instructions or responding adequately during intraoperative testing. Introducing the test structure preoperatively improves familiarity, which can in turn reduce patient anxiety and enhance cooperation during the surgery.

It is also important to consider that every intraoperative test adds to surgical time, which can cause discomfort and eventually increase the risk of the procedure. Therefore, the surgical team must balance the need for a comprehensive functional assessment to ensure functional preservation, while limiting the total duration of surgery as much as possible.

Lastly, the anesthesia regimen must be chosen carefully depending on the procedure and patient characteristics (Morshed et al. 2020): the “asleep–awake–asleep” protocol provides a deeper sedation during the craniotomy but requires more time to awaken the patient, who may experience fluctuations in consciousness. Alternatively, the “awake–awake–awake” approach uses only local anesthesia during craniotomy, therefore avoiding drowsiness during the intraoperative testing; however, this technique requires an especially cooperative patient.

## 6 New Perspectives for Language Mapping

Language mapping is constantly advancing, offering new and exciting possibilities for restoring communication in patients with neurological impairments and preventing some postoperative deficits. The loss of speech due to neurological injury often leads to devastating social isolation, significantly hindering a patient's ability to communicate quickly, precisely, and naturally (Peters et al. 2015). However, recent advancements in speech decoding technology have demonstrated the potential to translate brain activity into meaningful communication, even for individuals with paralysis.

Multimodal neuroprosthesis studies have successfully utilized ECoG to decode cortical articulatory activity into multiple real‐time output modalities, including text and speech audio synchronized with audiovisual (facial avatar and facial expressions) (Metzger et al. 2023). High‐density recordings of cortical activity in the speech‐production areas of the sensorimotor cortex have allowed researchers to decode full words and sentences in real time from individuals with paralysis or anarthria (Moses et al. 2021). Additionally, a new ECoG‐to‐speech framework has been developed, using a low‐dimensional intermediate representation generated by a specific pretraining on speech signals (Chen et al. 2024). This innovative framework decodes ECoG signals into interpretable speech acoustic parameters, such as pitch and voice frequencies, and can also synthesize these into a spectrogram, closely mimicking the speaker's own voice. The publicly available decoding framework (https://github.com/flinkerlab/neural_speech_decoding) is compatible with various deep learning model architectures and supports real‐time applications for brain–computer interfaces (BCIs), marking a major step forward in the field of language mapping and communication restoration.

Speech BCIs hold the potential to restore rapid communication for individuals with locked‐in syndrome, specifically after a brainstem tumor or stroke, by decoding neural activity associated with attempted speech into text or sound. These systems have demonstrated accurate decoding from small cortical regions, with detailed articulatory representation of phonemes that can persist even years after paralysis (Willett et al. 2023). These results offer a promising path forward in restoring communication for those who have lost the ability to speak due to a tumor or stroke. A recent study reported an intracortical speech neuroprosthesis capable of accessing a comprehensive 125,000‐word vocabulary with minimal training data requirements (Card et al. 2024). A patient with advanced amyotrophic lateral sclerosis (ALS) and severe dysarthria was able to achieve high accuracy in brain‐to‐text communication, with word error rates consistently below 5%. The device enabled the patient to communicate with family, healthcare professionals, and colleagues starting from the first day of use, highlighting the potential of speech neuroprostheses to provide immediate and meaningful communication benefits.

Looking ahead, speech decoding approaches have the potential to greatly enhance communication abilities and improve the autonomy of individuals with severe speech and neurological impairments.

## 7 Conclusion

The development and integration of advanced techniques, including stimulation‐based methods such as DES and recording‐based ones like ECoG, have significantly improved the precision and outcomes of brain surgeries, particularly for language mapping. Although these techniques differ in their methods and applications, they both play crucial and complementary roles in identifying eloquent cortical areas and preventing possible deficits during and after surgical interventions. DES remains the gold standard method for providing direct evidence of functional disturbance while carrying risks such as induced seizures during surgery. In contrast, ECoG offers an invasive alternative that records real‐time brain activity with high spatial and temporal resolution, without the risk of inducing seizures, making it a valuable tool for language mapping whenever it is available to the team and appropriate for the patient. The future of language mapping is promising, with emerging technologies in speech decoding through ECoG further expanding the potential for restoring communication for individuals with neurological impairments.

Here, we provide an overview of current methods of intraoperative language assessment in awake surgeries, comparing the gold standard stimulation technique DES, with the emerging recording‐based technology of ECoG and exploring their possible future applications. By synthesizing key findings from the literature, we aim to offer a valuable resource for researchers and clinicians in the field who seek to advance language mapping techniques and improve surgical outcomes and postoperative QoL in patients.

## Author Contributions

**Patricia Silva de Camargo**: conceptualization, writing – original draft, methodology, writing – review and editing, visualization, investigation, project administration, funding acquisition, resources, supervision. **Giovanna de Oliveira Santos e Souza**: conceptualization, visualization, writing – original draft, methodology, investigation, writing – review and editing, funding acquisition, resources. **Analía Arévalo**: conceptualization, writing – original draft, methodology, investigation, project administration, writing – review and editing, supervision, funding acquisition, resources. **Guilherme Lepski**: conceptualization, supervision, project administration, writing – review and editing, resources, funding acquisition, methodology, investigation.

## Consent

The authors have nothing to report.

## Ethics Statement

The authors have nothing to report.

## Conflicts of Interest

The authors declare no conflicts of interest.

## Peer Review

The peer review history for this article is available at https://publons.com/publon/10.1002/brb3.70900.

## Data Availability

Data sharing is not applicable to this article, as no new data were created or analyzed in this study.
